# Carbohydrate recognition and complement activation by rat ficolin-B

**DOI:** 10.1002/eji.201040612

**Published:** 2010-10-27

**Authors:** Umakhanth Venkatraman Girija, Daniel A Mitchell, Silke Roscher, Russell Wallis

**Affiliations:** 1 Department of Infection, Immunity and Inflammation, University of LeicesterLeicester, UK; 2 Clinical Sciences Research Institute, University of WarwickWarwick, UK; 3 Department of Biochemistry, University of LeicesterLeicester, UK

**Keywords:** Complement, Innate immunity, Lectin pathway

## Abstract

Ficolins are innate immune components that bind to PAMPs and structures on apoptotic cells. Humans produce two serum forms (L- and H-ficolin) and a leukocyte-associated form (M-ficolin), whereas rodents and most other mammals produce ficolins-A and -B, orthologues of L- and M-ficolin, respectively. All three human ficolins, together with mouse and rat ficolin-A, associate with mannan-binding lectin-associated serine proteases (MASPs) and activate the lectin pathway of complement on PAMPs. By contrast, mouse ficolin-B does not bind MASPs and cannot activate complement. Because of these striking differences together with the lack of functional information for other ficolin-B orthologues, we have characterized rat ficolin-B, and compared its physical and biochemical properties with its serum counterpart. The data show that both rat ficolins have archetypal structures consisting of oligomers of a trimeric subunit. Ficolin-B recognized mainly sialyated sugars, characteristic of exogenous and endogenous ligands, whereas ficolin-A had a surprisingly narrow specificity, binding strongly to only one of 320 structures tested: an *N*-acetylated trisaccharide. Surprisingly, rat ficolin-B activated MASP-2 comparable to ficolin-A. Mutagenesis data reveal that lack of activity in mouse ficolin-B is probably caused by a single amino acid change in the putative MASP-binding site that blocks the ficolin-MASP interaction.

## Introduction

Ficolins are pattern-recognition molecules of the innate immune system that target microbial surfaces and apoptotic cells [1, 2]. They are characterized by an N-terminal collagenous domain and C-terminal fibrinogen-like domain and assemble into oligomers of a subunit composed of three identical polypeptide chains. Humans produce two serum ficolins (L- and H-, also known as ficolins-2 and -3) together with M-ficolin (or ficolin-1), which has been localized to the surface of monocytes and identified in the secretory granules of neutrophils, monocytes and lung epithelial cells, as well as in serum at low concentrations [3, 4]. Rodents and pigs produce only two ficolins: ficolins-A and -B. Based on their primary structures and chromosomal environments, ficolin-A is the orthologue of human L-ficolin and ficolin-B corresponds to human M-ficolin [5]. Nevertheless, there are differences in the patterns of gene expression, for example, porcine ficolin-B is synthesized, stored, and secreted by neutrophils, but not by peripheral blood monocytes or platelets, whereas mouse ficolin-B is mainly expressed in the bone marrow and spleen [6], as well as by peritoneal macrophages [7].

Human L-ficolin binds to acetylated compounds, lipoteichoic acid, a major component of the cell walls of Gram-positive bacteria and β-d-glucan, a component of yeast cell surfaces [8–11]. It has several binding sites on each fibrinogen-like domain: an outer binding pocket, also present in H-and M-ficolins, together with three unique sites, which form a continuous binding surface for various acetylated and neutral carbohydrate ligands. L-ficolin binds to *Salmonella typhimurium*, group B streptococci and to various serotypes of *S. pneumoniae* and *Staphylococcus aureus* [12]. It also participates in the clearance of apoptotic and dead host cells [13]. Relatively little is known about the binding specificity of H-ficolin. It has been shown to bind to some carbohydrate ligands, including d-fucose, and galactose, but failed to recognize any of the ligands in a large glycan screen, suggesting that carbohydrates are not its major target [8, 14]. Nevertheless, it has been shown to bind to the Gram-positive bacterium *Aerococcus viridians* [15]*.* M-ficolin binds to acetylated sugars including sialic acid containing structures and has been shown to target S. *aureus* and *S. typhimurium*. Less is known about the sugar specificities of ficolins from other species, although mouse ficolins-A and -B bound to *N*-acetylglucosamine (GlcNAc) and *N*-acetylgalactosamine when conjugated to BSA and ficolin-B also bound conjugated sialic-acid containing structures [16].

All three human ficolins are capable of associating with mannan-binding lectin (MBL)-associated serine proteases (MASPs) and activating the lectin branch of the complement cascade when they bind to microbial targets [17]. MASP-2 alone is sufficient to activate the lectin pathway by cleaving C4 and then C2-bound C4b to form the C3 convertase, the central enzyme in the complement cascade [18]. Recently, MASP-1 has been shown to cleave factor D, a key enzyme in the alternative pathway [19]. It also activates human endothelial cells *via* protease-activated receptor-4 and in this way regulates endothelia function and may contribute towards development of the inflammatory response [20].

In contrast to human M-ficolin, mouse ficolin-B neither binds MASP nor activates complement [16]. Furthermore, it colocalized with lysosomal markers in peritoneal macrophages suggesting that it may be secreted upon activation of these cells, or act as an intracellular scavenger to target and facilitate clearance of PAMP-bearing debris [7]. To determine if lack of complement activity is unique to mouse ficolin-B or is common in this class of ficolins, we have characterized rat ficolin-B, which shares 79 and 88% identity with its human and mouse othologues, but has not been examined previously. The data show that rat ficolin-B recognizes ligands found on self-structures as well as tumour cells and pathogens. Furthermore, it can activate complement with similar activity to ficolin-A, and thus functionally more closely resembles its human, rather than mouse, counterpart.

## Results

### Expression and characterization of rat ficolin-B

**Figure 1:**
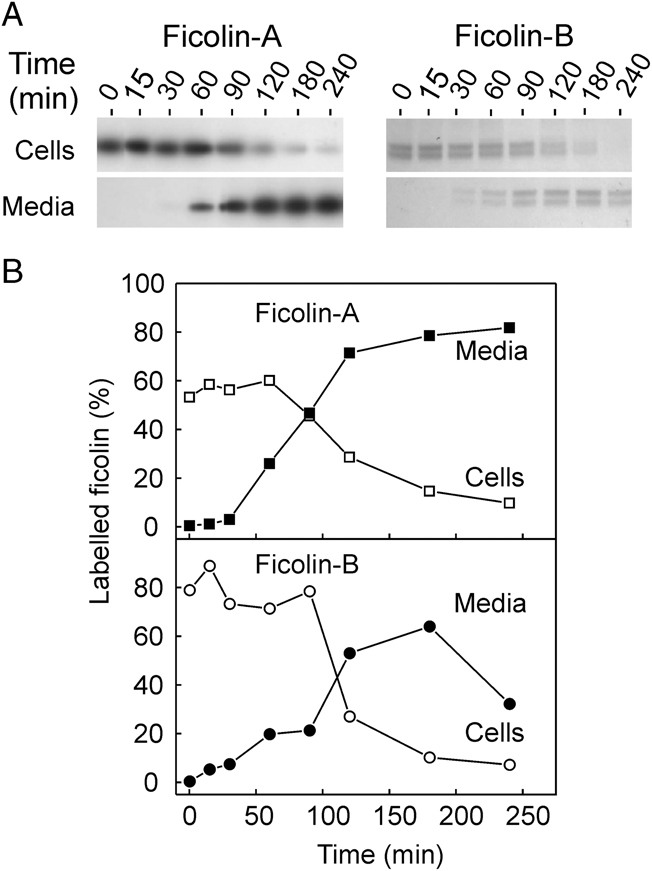
Secretion of rat ficolins-A and -B. (A) ^35^S-Pulse-labelled ficolins from the intracellular and secreted fractions of transfected CHO cells were pelleted using GlcNAc Sepharose and analyzed by SDS-PAGE. Labelled ficolin was quantified by scanning autoradiographs. (B) Time-course of secretion for ficolins-A and -B. Data are expressed relative to the total amount of label incorporated.

To produce pure ficolin-B, free of contamination by ficolin-A or other serum lectins, recombinant protein was produced using a mammalian expression system. Initially, it was not clear whether ficolin-B would be secreted from producing cells or retained internally, so studies were carried out to follow the kinetics of biosynthesis, using ficolin-A for comparison. In these experiments, transfected CHO cells were incubated for a brief pulse of ^35^S-labelled methionine, followed by extended incubation in unlabelled media. By comparing the amounts of labelled protein in the intracellular and extracellular fractions over time, the kinetics of biosynthesis and secretion could be followed. As shown in Fig. 1, labelled ficolin-B appeared in the culture medium of producing cells soon after the initial pulse and was secreted with a half-time of ∼110 minutes. There was no evidence of retention of ficolin-B within cells, nor of degradation. The secretion rate is typical of many secreted proteins, including rat MBL-A and MBL-C (Heise *et al.* 2000) and was only slightly slower than ficolin-A (half-time ∼90 minutes). Thus, any intracellular retention signals present in the ficolin-B polypeptide are not recognized by CHO cells.

**Figure 2:**
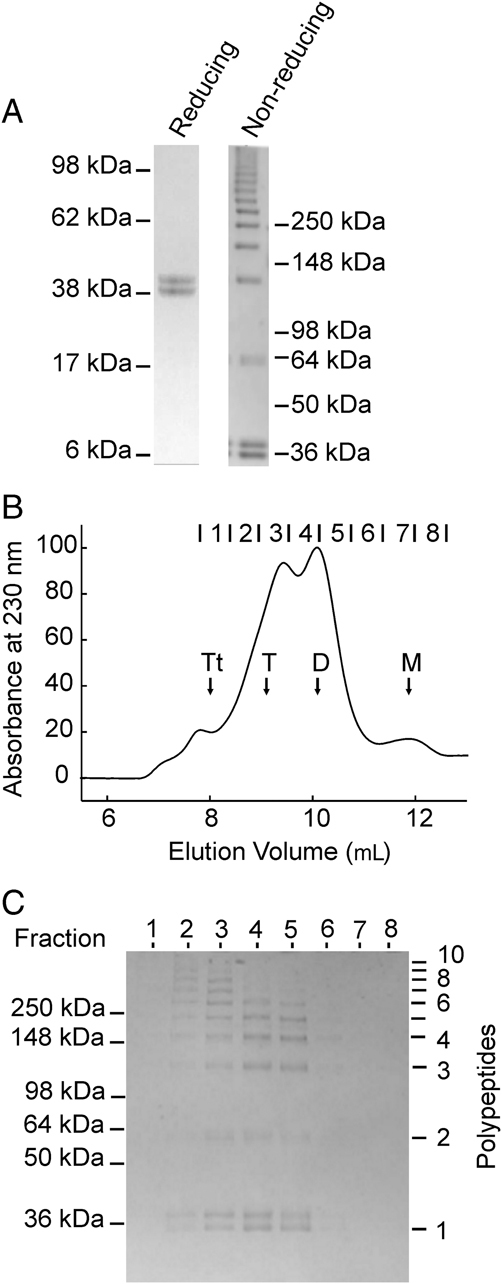
Oligomeric structure of rat ficolin-B. (A) Purified ficolin-B separated by SDS-PAGE (4–12% gradient gel) under reducing and non-reducing conditions. Proteins were stained using Coomassie blue. (B) Gel filtration of ficolin-B on a Superdex-200 column. The elution positions of monomers (M), dimers (D), trimers (T) and tetramers (Tt) of rat ficolin-B are indicated. (C) Fractions collected across the gel filtration peaks were separated by gel electrophoresis on a 4–12% gradient SDS-polyacrylamide gel under non-reducing conditions, and proteins were stained with Coomassie blue.

Ficolin-B was harvested from the culture medium of producing cells and purified by affinity chromatography on GlcNAc-Sepharose columns. Yields from several different cell-lines were relatively low, with only 50–100 μg of purified protein *per* litre of culture medium, compared to 4–5 mg/L of ficolin-A [21]. Purified ficolin-B migrated as two bands on SDS-polyacrylamide gels under reducing conditions, with apparent molecular masses of 39 and 40 kDa (Figs. 1 and 2), both of which are greater than the molecular mass of 34 kDa calculated from the amino acid sequence. There is one potential N-linked glycosylation site in the fibrinogen domain and five potential O-linked sites in the collagen-like domain, so the two species observed on gels probably reflect different glycoforms. N-terminal sequencing of each band yielded the sequence: ADTCPEVKVL, indicating that the signal peptide is cleaved between Ala22 and Ala23 of the synthesized polypeptide during biosynthesis.

Under non-reducing conditions, ficolin-B migrated as mixtures of covalently linked polypeptides on SDS-polyacrylamide gels ranging from one to more than 12 chains, so most polypeptides must be linked by interchain disulfide bonds (Fig. 2). It eluted as several overlapping peaks from a gel filtration column, signifying the presence of different oligomeric forms. Oligomers were identified by comparison with the elution positions of the different forms of ficolin-A, which have comparable sizes and have been characterized previously [21]. In this way, two major oligomers were identified consisting of six and nine polypeptides (subsequently called dimers and trimers of subunits), together with smaller amounts of monomers and tetramers of subunits. Trace amounts of even larger oligomers were observed, probably comprising pentamers and/or hexamers of subunits. Analysis of fractions collected across the elution profile confirmed that the disulphide-bonding pattern in oligomers is heterogeneous (Fig. 2C). For example, fraction 3 comprises mainly trimers of subunits, but a ladder of covalently linked polypeptides (from 1 to 9) is observed on SDS gels under non-reducing conditions, indicating that the different forms must be assembled from combinations of covalently linked polypeptides, associated through non-covalent interactions. Overall, the oligomeric composition is broadly similar to rat ficolin-A, which also comprises mixtures of disulphide-linked oligomers [21].

### Carbohydrate specificity of rat ficolins

**Figure 3:**
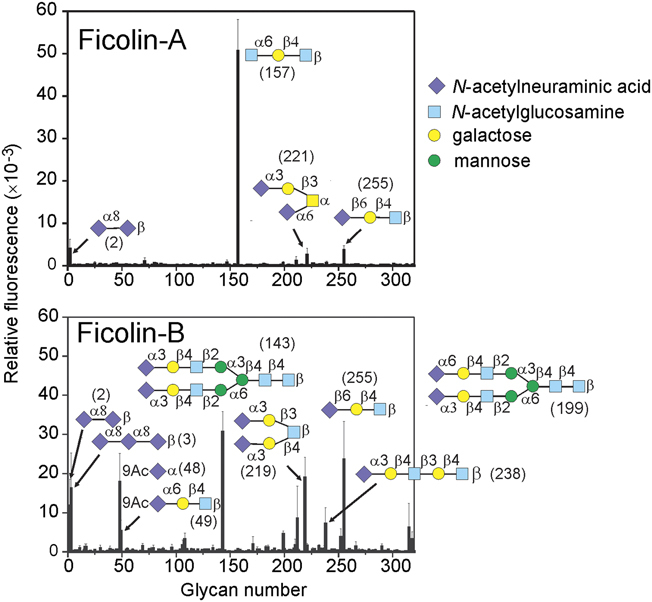
Glycan array screening of rat ficolins-A and -B. Fluorescently labelled ficolins (200 μg/mL) were used to probe the glycan array comprising 320 ligands. Error bars represent the means±SD of six measurements. Structures of the best glycan ligands are indicated.

Because little detailed information is known about the binding specificities of rat ficolins, we examined their lectin properties towards a broad screen of endogenous and exogenous carbohydrates using the Core H Glycan array technology facility at The Consortium for Functional Glycomics, Emory University (GA, USA). Ficolins were labelled with a fluorescent dye and used to probe a library of 320 ligands (Supporting Information). Ficolin-A is known to bind to *N*-acetylated ligands such as GlcNAc [21], but specificity towards natural ligands has not been examined. Surprisingly, it bound appreciably to only one of the ligands tested: a trisaccharide containing a terminal α1-6 linked GlcNAc residue (ligand 157) (Fig. 3). No binding was observed towards ligands lacking the terminal GlcNAc (ligand 153) or when the GlcNAc was linked *via* α1-3 (ligand 156), β1-2 (ligand 158), β1-3 (ligand 165) or β1-6 (ligand 176) glycosidic linkages (Table 1). Thus, the type and mode of attachment of the terminal sugar residue is critical. Although the binding signal from the next best ligand was>10-fold lower, certain sialyated sugars and a disialic acid also bound above background level, neither of which have been previously identified as ligands for a ficolin-A orthologue. Notably, all ligands contained multiple N-acetyl groups, suggesting that internal acetylated residues may be recognized by ficolin-A, in addition to the terminal moiety.

**Table 1:** Binding of ficolin-A to selected carbohydrate structures

Ligand number	Ligand	Binding signal	Standard error
157	GlcNAcα1-6Galβ1-4GlcNAcβ-	50680	7409
*Non-ligands*			
153	Galβ1-4GlcNAcβ-	33	24
156	GlcNAcα1-3Galβ1-4GlcNAcβ-	93	29
158	GlcNAcβ1-2Galβ1-3GalNAcα-	155	68
165	GlcNAcβ1-3Galβ1-4GlcNAcβ-	99	12
176	GlcNAcβ1-6Galβ1-4GlcNAcβ-	84	30
*Sialated ligands*			
2	Neu5Acα2-8Neu5Acβ-	4003	2270
255	Neu5Acβ2-6Galβ1-4GlcNAcβ-	3714	1022
221	Neu5Acα2-3Galβ1-3(Neu5Acα2-6)GalNAcα-	2507	1622

In contrast to ficolin-A, rat ficolin-B bound to a number of ligands in the glycan screen (Table 2), with a marked preference for sialyated structures with terminal *N*-acetylneuraminic but not *N*-glycolyneuraminic acid (a sialic acid found in many mammals but not humans; ligands 257–264, Supporting Information). Moreover mono- and biantennary sialated sugars with α2-3 linkages but not α2-6 linkages were preferentially recognized (for example, compare ligands 143, 199 and 54 (Table 2)). Ligands could be divided into four main groups: di- and trisialic acids; mono- or biantennary structures containing the trisaccharide sialyl-*N*-acetyllactosamine (Sialyl-Gal-GlcNAc), 9-acetylated sialated ligands and more weakly to certain fucosylated structures. It is notable that such sequences are commonly found on endogenous structures including cell surfaces and tumour cells, as well as on exogenous ligands including pathogenic microbes and viruses [22, 23].

**Table 2:** Binding of ficolin-B to selected carbohydrate structures

Ligand number	Ligand	Binding signal	Standard error
*Sialated ligands*			
143	Neu5Acα2-3Galβ1-4GlcNAcβ1-2Manα1-3(Neu5Acα2-3Galβ1-4GlcNAcβ1-2Manα1-6)Manβ1-4GlcNAcβ1-4GlcNAcβ-	30636	5205
255	Neu5Acβ2-6Galβ1-4GlcNAcβ-	23573	9774
219	Neu5Acα2-3Galβ1-3(Neu5Acα2-3Galβ1-4)GlcNAcβ-	18985	5184
238	Neu5Acα2-3Galβ1-4GlcNAcβ1-3Galβ1-4GlcNAcβ-	7170	4094
199	Neu5Acα2-6Galβ1-4GlcNAcβ1-2Manα1-3(Neu5Acα2-3Galβ1-4GlcNAcβ1-2Manα1-6)Manβ1-4GlcNAcβ1-4GlcNAcβ-	4591	734
318	Neu5Acα2-3Galβ1-4GlcNAcβ1-2Manα1-3(Neu5Acα2-6Galβ1-4GlcNAcβ1-2Manα1-6)Manβ1-4GlcNAcβ1-4GlcNAcβ-	3263	1911
221	Neu5Acα2-3Galβ1-3(Neu5Acα2-6)GalNAcα-	1528	661
232	Neu5Acα2-3Galβ1-4(Fucα1-3)GlcNAcβ1-3Galβ-	1415	422
*Non-ligand*			
54	Neu5Acα2-6Galβ1-4GlcNAcβ1-2Manα1-3(Neu5Acα2-6Galβ1-4GlcNAcβ1-2Manα1-6)Manβ1-4GlcNAcβ1-4GlcNAcβ-	78	53
*9-Acetylated sialated ligands*			
48	9NAcNeu5Acα-	17882	7296
49	9NAcNeu5Acα2-6Galβ1-4GlcNAcβ-	5319	2738
*Di- and trisialic acids*			
3	Neu5Acα2-8Neu5Acα2-8Neu5Acβ-	16240	8995
2	Neu5Acα2-8Neu5Acβ-	11811	6744
252	Neu5Acα2-8Neu5Acα-	3731	2206
*Fucosylated ligands*			
108	Galα1-4(Fucα1-2)Galβ1-4GlcNAcβ-	3180	1576
69	Fucα1-2Galβ1-4GlcNAcβ1-3Galβ1-4GlcNAc-	1477	692
290	Galα1-3(Fucα1-2)Galβ-	1350	720

### Complement activation

**Figure 4:**
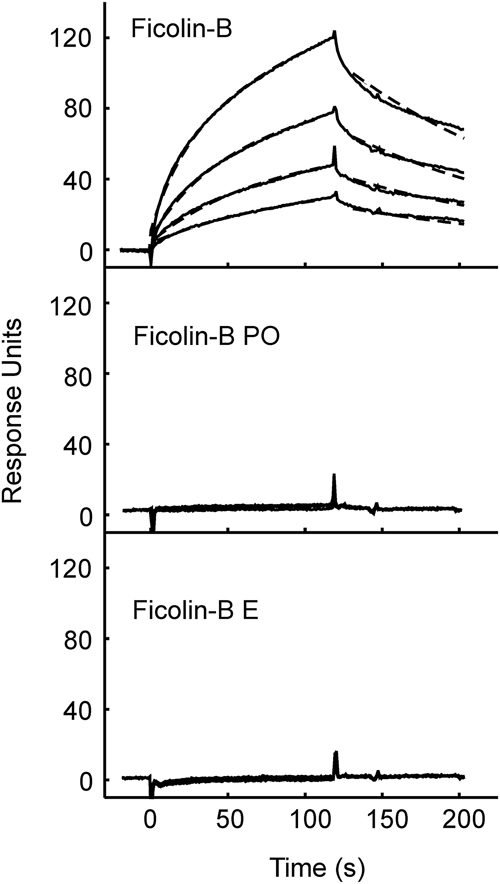
MASP-2A binding to immobilized ficolin-B and ficolin-B mutants using surface plasmon resonance. Equivalent amounts of ficolin-B and ficolin-B mutants (5000–6000 RU) were immobilized on to a CM5 sensor chip and MASP-2A was injected at 0.04, 0.08, 0.16 and 0.33 μM. The dotted line shows the fit to a two-binding site model for ficolin-B. In ficolin-B PO, Lys54 and Ala55 have been mutated to proline and hydroxyproline respectively. In ficolin-B E, Ala55 was replaced by a glutamic acid residue.

Rat ficolin-A binds MASP and can activate complement [21], but the properties of ficolin-B are not known. To test binding to MASP-1 and MASP-2, ficolin-B was immobilized on a sensor chip and interactions with MASP were monitored using surface plasmon resonance. Both MASP associated with ficolin-B in the presence of Ca^2+^ ions to form relatively stable complexes. As with ficolin-A, the binding kinetics were complex. The data fitted best to a two-reaction parallel binding model for MASP-2A (Fig. 4), with apparent equilibrium dissociation constants *K*_*D*1_ and *K*_*D*2_ of 752 and 23 nM. Association rate constants (*k*_on_) were 1×10^4^ and 2.6×10^5^ M^−1^s^−1^ and dissociation rate constants (*k*_off_) were 5.8×10^−3^ and 6×10^−3^ s^−1^, respectively. The kinetics of MASP-1 binding was similar to MASP-2 (data not shown). *K*_*D*1_ and *K*_*D*2_ were 392 and 8.4 nM, with *k*_on_ values of 2.4×10^4^ and 5.7×10^5^ M^−1^s^−1^ and *k*_off_ values of 9.1×10^−3^ and 4.3×10^−3^ s^−1^, respectively. The biphasic kinetics are typical of rat MBL-MASP and ficolin-MASP interactions and probably reflect binding of a second MASP to ficolin oligomers at high MASP concentrations, although 1:1 complexes predominate under physiological concentrations [24]. No binding of MASP-1 or MASP-2 was detected in the presence of EDTA, confirming that the interactions are Ca^2+^ dependant (data not shown). Overall, the binding data were broadly comparable to the interactions of ficolin-A with MASP-2 [21], but markedly different from mouse ficolin-B, which does not bind MASPs despite a high degree of sequence identity [16].

**Figure 5:**
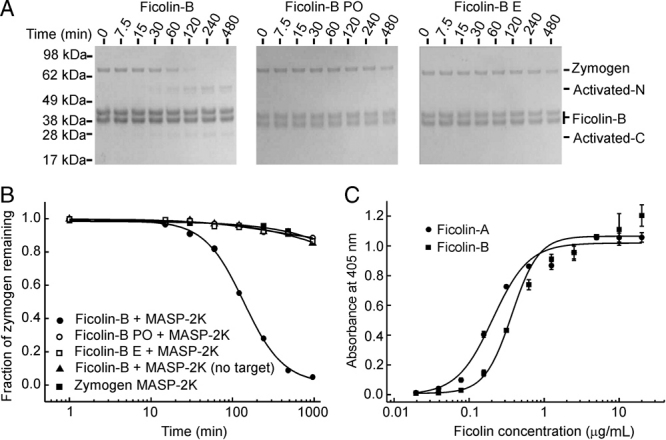
Kinetics of MASP-2 activation by ficolin-B and ficolin-B mutants. Activation of MASP-2K by WT and mutant ficolins on GlcNAc-Sepharose as an activating target. (A) Aliquots of protein complexes taken at different time points were analyzed by SDS-PAGE (10% gel), stained with Coomassie blue. The migration positions of zymogen MASP-K and the N- and C-terminal fragments of activated MASP-2K (activated –N and –C) are indicated. (B) The amount of activation was determined by measuring cleavage of zymogen MASP-2K. Only 12% cleavage was observed for either mutant after 16 h comparable to the amount of autoactivation of MASP-2K alone. In ficolin-B PO, Lys54 and Ala55 have been mutated to proline and hydroxyproline, respectively. In ficolin-B E, Ala55 was replaced by a glutamic acid residue. (C) Complement activation by recombinant rat ficolins-A and -B. Complement activation was measured on acetylated-BSA using an alkaline phosphatase-conjugated antibody specific for the membrane attack complex, following incubation of chromogenic substrate. Background absorbance, in the absence of ficolin, was subtracted from the data. Data are the average±SE from duplicate observations.

MASP-2 in complex with MBL or ficolin is necessary and sufficient to activate the lectin pathway of complement activation, so we next examined if rat ficolin-B could activate complement. Ficolin-B triggered MASP-2K activation, using GlcNAc-Sepharose as a target, with a half-time of ∼110 min (Fig. 5; compared to 56 min by ficolin-A under comparable conditions (data not shown)). Very little activation was observed in the absence of GlcNAc-Sepharose (∼10% over 16 h), and this was no faster than auto-activation of zymogen MASP-2K alone. Thus, MASP-2 activation is both target and ficolin dependent (Fig. 5B). To confirm that MASP activation by ficolin-B leads to complement activation, we measured deposition of the membrane attack complex, on acetylated BSA as an activating target. Addition of recombinant ficolin-B to human serum depleted of endogenous MBL and ficolins activated complement in a concentration-dependent manner (Fig. 5C) and with similar activity to ficolin-A. Thus, rat ficolin-B, unlike its mouse counterpart, activates the lectin pathway of complement *via* a comparable mechanism to ficolin-A (and MBL) in which the ficolin increases the rate of MASP-2 autolysis, but only when complexes bind to a target surface.

MASPs bind to a characteristic motif on the collagenous domain of MBL and ficolins comprising the sequence Hyp-Gly-Lys-Xaa-Gly-Pro, in which Xaa typically represents a hydrophobic aliphatic residue or a methionine, and the lysine residue is essential for binding [21, 24, 25]. To confirm that MASP bind to the equivalent site in rat ficolin-B, Lys54 and Ala55 within the sequence Hyp-Gly-Lys-Ala-Gly-Pro were changed to proline and hydroxyproline residues, respectively (K54P, A55O). The resulting mutant (Ficolin-B PO) was expressed and purified from CHO cells and resembled WT protein upon gel filtration chromatography and SDS-PAGE (data not shown), confirming that it assembles correctly during biosynthesis. However, it failed to bind MASP-1 or MASP-2 (Fig. 4) and did not activate MASP-2 (Fig. 5), demonstrating that Lys54 and/or Ala55 form part of the MASP-binding site. Although mouse ficolin-B possesses a similar binding motif, it is unusual compared to other ficolins because it contains an acidic residue adjacent to the key lysine: Hyp-Gly-Lys-**Glu**-Gly-Pro. In previous work, introduction of a glutamate into rat ficolin-A reduced the affinity of MASP-binding and MASP-2 activation by ∼7-fold, so it is somewhat disruptive [21]. To examine the effect of such a mutation within the ficolin-B framework, we made a comparable change in the rat protein (ficolin-B E). The resulting mutant failed to bind to MASP-1 or MASP-2 at the highest concentrations tested (0.5 μM; Fig. 4), despite having equivalent structural and lectin properties to WT ficolin-B. It also failed to activate MASP-2, even after prolonged incubation (up to 16 h), indicating that the glutamate residue disrupts complement activation by ficolin-B by preventing association with MASP-2 (Fig. 5). Thus, mouse ficolin-B differs from its human and rat counterparts probably as a result of a single amino acid substitution in the collagenous domain.

## Discussion

This study indicates that rat ficolin-B binds MASP and activates MASP-2 on a target surface, to trigger complement activation. The rate of MASP-2 activation was only twofold lower than for rat ficolin-A, so ficolin-B-mediated complement activation is likely to be an important physiological activity for neutralizing bacteria. This work also provides an explanation for the lack of complement activity of mouse ficolin-B: a glutamate residue in place of the usual aliphatic/hydrophobic residue in the residual MASP-binding site. Substitution of a glutamate for the natural alanine residue in rat ficolin-B completely abolished MASP binding and complement activation, so the acidic residue is particularly disruptive within the ficolin-B scaffold, compared to ficolin-A. Mouse ficolin-B is probably unusual in its lack of complement function, because other ficolins for which sequences have been determined contain archetypal MASP-binding sites [21]. This difference is particularly pertinent for studies using mouse models of lectin pathway function.

The ligand specificity of rat ficolin-B is distinct from that of ficolin-A, so each protein targets a different set of physiological ligands. The “best” ligand of ficolin-A (157, which contains a terminal α1-6 GlcNac moiety) is not commonly found in mammalian glycans, consistent with its ability to recognize foreign structures. Some sialated structures were also recognized, but these all bound relatively weakly. Moreover, ligand 221 (Table 1) is also atypical of mammalian structures, and the disialic acid (ligand 2, also recognized by ficolin B) is found on pathogenic bacteria [26]. In contrast, Ficolin-B exclusively recognized terminally sialylated ligands. Specificity was restricted to α2-3, β2-6 and α2-8 linkages, rather than α2-6 linkages. As well as being found on the surface of a variety of pathogenic microbes, viruses and tumour cells, these motifs are likely to be markers of normal host cells [22]. For example, certain pathogenic bacteria (*e.g. Escherichia coli* K1 and *Neisseria meningitidis*) express capsular polysialic acids that help evade the immune system, and these are chemically identical to sugars found on neural cell adhesion molecules [26]. Similarly, 9-O-acetylated sialic acids are commonly found in mammalian sugars, but are also present on pathogens, including parasitic protozoa and fungi [27, 28]. Given that sialylation is both species and tissue specific [29, 30], more detailed analysis of rat glycans is necessary to fully understand the physiological implications of the ligand specificity of rat Ficolin-B. Nevertheless, the likely binding preference for endogenous as well as exogenous sugar structures, raises a key unresolved question: how complement activation leading to self damage is avoided on host tissues? One possibility is that M-ficolin/ficolin-B are secreted only upon activation of producing leukocytes, leading to highly localized and/or rapid release and turnover. Once bound, they could neutralize their targets either through complement activation or *via* opsonophagocytosis. Consistent with the latter possibility, M-ficolin undergoes a conformational change at lower pH, as would be found in lysosomes, rendering it incapable of binding to its ligands [31]. While microbes or apoptotic cell debris are probably destroyed by the cell, residual M-ficolin may be recycled and deposited back into secretory granules for subsequent use. Although mouse ficolin-B lacks the ability to activate complement *via* the lectin pathway, it probably also functions as an opsonin to clear pathogens and or apoptotic debris. Notably, it is found in the lysosomes and/or late endosomes of macrophages, and its production is upregulated upon macrophage activation [7].

Overall, rat ficolin-B recognizes similar classes of ligands to human M-ficolin and mouse ficolin-B, but there are notable differences. For example, both rat and human proteins bound to sialylated sugars, but the former recognized a broad range of biantennary structures (*e.g.* ligands 143, 219, 255 and 199), whereas the latter recognized only one of these with substantial affinity: ligand 143. By contrast, human ficolin-M bound to several gangliosides [14], which were not targeted by rat ficolin-B.

A surprising finding is the narrow carbohydrate specificity of rat ficolin-A with only one of the targets binding appreciably (ligand 157). Although this was also one of the major ligands of human L-ficolin [9, 14], L-ficolin recognized a variety of other ligands that were not targeted by the rat protein. Although most of the residues that form the binding pockets are conserved in rat ficolin-A, serine residues in site S2 of human L-ficolin, which form hydrogen bonds *via* their hydroxyl groups to sugar ligands are replaced by more bulky threonine and asparagine residues, which might exclude certain ligands [14]. Another interesting difference is the weak but significant binding of certain sialyated structures by the rat but not the human protein. Unlike M-ficolin, L-ficolin does not bind sialyated sugars due to differences at the S1 binding pocket. Notably, the hydroxyl group of a tyrosine residue at position 271 in M-ficolin provides a direct hydrogen bond to *N*-acetylgalactosamine or sialic acid moieties, whereas the corresponding phenylalanine in L-ficolin cannot make the same contacts [14]. Rat ficolin-A possesses a tyrosine at this position so in principal could share some of the properties of M-ficolin, hence explaining its somewhat different ligand specificity. Thus, small differences in the largely conserved binding pockets probably dictate the subtle binding preferences of these defence lectins.

## Material and methods

### Cloning, expression and purification of rat ficolin-B

The cDNA of rat ficolin-B was amplified from a rat cDNA library kindly provided by Prof. Wilhelm Schwaeble (University of Leicester, UK) using PCR, and was cloned into pGEM-T cloning vector (Promega). The forward and reverse oligonucleotides were GAACTCGAGGCCACC**ATG**GTCCTGGGATCTGCT, where the ribosome-binding site is underlined and the start codon is in bold, and AGAGAATTC**CTA**GATGAGGCGCACCTTCAT, where the stop codon is in bold. The cDNA was cloned into a mammalian expression vector, pED4 [32], the sequence confirmed and the resulting construct was introduced into the CHO cell line DXB11. Production was achieved following amplification using the dihydrofolate reductase inhibitor, methotrexate [21]. Culture media were harvested and protein purified by affinity chromatography on GlcNAc-Sepharose columns (1-mL column for 250 mL of media) using the strategy described previously for ficolin-A [21]. We also attempted to express mouse ficolin-B using the same strategy. However, only trace amounts of protein was produced from transfected cells, so further analysis was not possible.

### Other protein components

Recombinant rat ficolin-A and modified forms of rat MASP-2 were produced in a CHO cell-expression system and purified as described previously by affinity chromatography on GlcNAc-Sepharose and nickel-Sepharose columns, respectively [21, 33]. WT rat MASP-1 and MASP-2 are toxic to producing CHO cells because they autoactivate during biosynthesis. We therefore used two modified forms of MASP-2, both of which are secreted as zymogens and have been characterized with regard to their structures, activation and catalytic properties [33, 34]. MASP-2A is a catalytically inactive form in which the active site serine at position 613 has been replaced by an alanine. MASP-2K is a catalytically active form, in which the arginine residue at the cleavage site for zymogen activation (Arg^424^) has been changed to a lysine residue to slow down the rate of spontaneous autoactivation during biosynthesis. A modified form of MASP-1, called MASP-1ent, was used for binding analysis [34]. This is a catalytically active form in which residues Lys^425^–Arg^429^, immediately preceding the cleavage site for zymogen activation, were replaced with the recognition sequence of the serine protease enterokinase: Asp-Asp-Asp-Asp-Lys. Binding and activation assays were carried out using MASP-2A or MASP-1ent and MASP-2K, respectively. Mutant forms of ficolins were created by PCR and expressed in the same way as the WT protein. All mutations were verified by sequencing of the entire cDNA, within the expression vector, prior to transfection of the CHO cells.

### N-terminal sequencing

Samples were separated on 10% Novex BIS-TRIS NuPAGE precast gels using 2-(*N*-morpholino)ethanesulfonic acid buffer in a Novex×Cell II Mini-Cell gel apparatus. The protein bands were electroblotted onto a Novex 0.2 μm polyvinylidene difluoride membrane (Invitrogen) using the Novex Blot module. The membrane was stained with Coomassie Brilliant Blue and target bands were excised, washed with 10% methanol and sequenced on a 494A Procise protein sequencer (Applied Biosystems, Warrington, UK) for ten cycles using the standard sequencing protocol.

### Pulse-chase labelling of ficolins

Pulse-chase experiments were carried out essentially as described in [35]. Briefly, CHO cells producing rat ficolin-A or -B were grown to confluence in 35×10-mm tissue culture dishes containing 2 mL of minimal essential medium-α, lacking nucleosides and supplemented with 10% dialyzed foetal calf serum and 0.5 μM methotrexate. Culture medium was changed daily for an additional 4 days. Before labelling, all culture media were pre-warmed to 37°C in 5% CO_2_. Cells were incubated with 2 mL of methionine-free medium for 5 min to diminish any intracellular methionine. Labelling was initiated with 1 mL of fresh methionine-free medium containing ^35^S-methionine (56 μCi/mL). After 15 min, the pulse was terminated by removing the ^35^S-methionine-containing medium and the cells were incubated with medium containing a 20-fold excess of unlabelled methionine. After various time intervals, cells were released from the culture dish by scraping, pelleted by centrifugation at 2000 rpm in a bench-top centrifuge, and disrupted by resuspension in cell lysis buffer (50 mM Tris-HCl, pH 7.4, containing 500 mM NaCl and 1% Triton X-100). GlcNAc-Sepharose (10 μL) was added to each fraction and mixtures were incubated on ice for 10 min. Bound ficolin was pelleted by centrifugation and separated by SDS-PAGE. Ficolins were quantified by scanning autoradiographs using a ChemiGenius (Syngene).

### Glycan array analysis

Alexa Fluor® 488 tetrafluorophenylcarboxylate ester (Invitrogen, Paisley, UK) was dissolved in DMSO to a final concentration of 1 mg/mL, and 20 μL was added to purified ficolin (250 μL of a 400 μg/mL solution) and incubated for 2 h at room temperature. Excess label was removed by dialysis. The labelled ficolin was then screened using the Glycan array technology facility at The Consortium for Functional Glycomics, Emory University. This facility uses printed glycan microarray chips with 320 different carbohydrate targets of natural and synthetic glycans attached to the chip *via* amino linkers. Binding was detected by measuring the fluorescence associated with each glycan. In each case, screening of the whole chip was performed with a total volume of 70 μL of 200 μg/mL ficolin in 20 mM HEPES, pH 7.4, with 140 mM NaCl, 5 mM CaCl_2_, and repeated six times. Error bars represent the average±SE.

### Surface plasmon resonance

Measurements were performed using a BIAcore 2000 instrument (GE Healthcare). Protein ligands were diluted into 10 mM sodium acetate, pH 4.0, and immobilized on to the carboxymethylated dextran surface of a CM5 sensor chip (GE Healthcare), using amine coupling chemistry. Binding was measured in 10 mM HEPES (pH 7.4) containing 150 mM NaCl and 5 mM CaCl_2_ at a flow rate of 5 μL/min and at 25°C. After injection of ligand, the protein surface was regenerated by injection of 10 μL of 10 mM HEPES buffer (pH 7.4) containing 1 M NaCl and 5 mM EDTA. Data were analyzed by fitting association and dissociation curves to Langmuir binding models for several protein concentrations simultaneously, using BIAevaluation 4.1 software (GE Healthcare). Increasingly complex models were tested until a satisfactory fit to the data was achieved. Apparent equilibrium dissociation constants (*K*_D_) were calculated from the ratio of the dissociation and association rate constants (*k*_off_/*k*_on_). Errors were determined from two separate experiments.

### MASP-2 activation assays

Activation was measured by following MASP autolysis as described previously [34]. Proteins were separated by SDS-PAGE under reducing conditions and the amount of MASP cleaved was quantified by densitometry. Data are the mean±SE from at least two separate experiments using different protein preparations, unless otherwise stated.

### Complement activation

Complement activation was measured using the Weislab Complement system MBL pathway kit (Euro-diagnostic). Assays were conducted as described, with the exception that wells of the microtitre dish were coated with acetylated-BSA (100 μL of 0.1 mg/mL), in 50 mM sodium bicarbonate (pH 9.6), overnight at 4°C, instead of mannan. Whole human serum was depleted of endogenous MBL and ficolins before setting up the assay by passage through two GlcNAc-Sepharose columns (0.5 for 1 mL of serum). Data represent the average±error from duplicate observations.

## Abbreviations

GlcNAc: *N*-acetylglucosamine
MASP: MBL-associated serine protease
MBL: mannan-binding lectin
