# Supplementary material for: Carbohydrate recognition and complement activation by rat ficolin-B
Source: Eur J Immunol. 2010 Oct 27;41(1):214–23. doi: 10.1002/eji.201040612 (PMC3179595; doi:10.1002/eji.201040612)
Supplement: Supplementary file 1 [file eji0041-0214-SD1.pdf]

European Journal of Immunology

Supporting Information

for

DOI 10.1002/eji.201040612

Carbohydrate recognition and complement activation by rat ficolin-B

Umakhanth Venkatraman Girija, Daniel A. Mitchell, Silke Roscher and Russell Wallis

© 2010 WILEY-VCH Verlag GmbH & Co. KGaA, Weinheim

Glycan binding by rat ficolins-A and -B.

Details of spacer arms (Sp) can be found at http://www.functionalglycomics.org

		Ficolin-A		Ficolin-B	
Glycan number	Glycan name	Average binding	Standard error	Average binding	Standard error
1	Neu5Acα2-8Neu5Acα-Sp8	46	47	10	10
2	Neu5Acα2-8Neu5Acβ-Sp17	4003	2270	11811	6744
3	Neu5Acα2-8Neu5Acα2-8Neu5Acβ-Sp8	92	32	16240	8995
4	Neu5Gcβ2-6Galβ1-4GlcNAc-Sp8	25	15	39	78
5	Galβ1-3GlcNAcβ1-2Manα1-3(Galβ1-3GlcNAcβ1-2Manα1-6)Manβ1-4GlcNAcβ1-4GlcNAcβ-Sp19	19	3	-26	10
6	Neu5Acα2-6Galβ1-4GlcNAcβ1-2Manα1-3(Neu5Acα2-6Galβ1-4GlcNAcβ1-2Manα1-6)Manβ1-4GlcNAcβ1-4GlcNAcβ-Sp12	197	160	281	88
7	α-D-Gal–Sp8	156	43	399	201
8	α-D-Glc–Sp8	165	48	4	22
9	α-D-Man–Sp8	117	44	314	102
10	α-GalNAc–Sp8	237	40	43	38
11	α-L-Fuc–Sp8	222	179	965	688
12	α-L-Fuc–Sp9	128	71	5	27
13	α-L-Rhα–Sp8	-44	18	44	84
14	α-Neu5Ac–Sp8	-28	22	52	23
15	α-Neu5Ac–Sp11	105	33	390	424
16	β-Neu5Ac-Sp8	46	19	1279	767
17	β-D-Gal–Sp8	15	12	1204	872
18	β-D-Glc–Sp8	276	8	-20	3
19	β-D-Man–Sp8	80	31	178	70
20	β-GalNAc–Sp8	133	46	-9	6
21	β-GlcNAc–Sp0	184	78	196	186
22	β-GlcNAc–Sp8	201	38	-5	4
23	β-GlcN(Gc)-Sp8	149	51	48	41
24	(Galβ1-4GlcNAcβ)$_2$-3,6-GalNAcα–Sp8	84	48	19	30
25	GlcNAcβ1-3(GlcNAcβ1-4)(GlcNAcβ1-6)GlcNAc-Sp8	497	441	478	201
26	[3OSO3][6OSO3]Galβ1-4[6OSO3]GlcNAcβ-Sp0	-25	16	46	10
27	[3OSO3][6OSO3]Galβ1-4GlcNAcβ-Sp0	-4	28	41	80
28	[3OSO3]Galβ1-4Glcβ-Sp8	-17	9	-23	37
29	[3OSO3]Galβ1-4(6OSO3)Glcβ–Sp0	177	41	-29	11
30	[3OSO3]Galβ1-4(6OSO3)Glcβ–Sp8	413	213	956	705
31	[3OSO3]Galβ1-3(Fucα1-4)GlcNAcβ–Sp8	141	87	52	14
32	[3OSO3]Galβ1-3GalNAcα–Sp8	88	85	144	85
33	[3OSO3]Galβ1-3GlcNAcβ–Sp8	224	189	-14	16
34	[3OSO3]Galβ1-4(Fucα1-3)GlcNAcβ–Sp8	110	40	-9	8
35	[3OSO3]Galβ1-4[6OSO3]GlcNAcβ-Sp8	292	193	46	80
36	[3OSO3]Galβ1-4GlcNAcβ–Sp0	58	3	91	66
37	[3OSO3]Galβ1-4GlcNAcβ-Sp8	35	26	244	190
38	[3OSO3]Galβ–Sp8	-12	11	40	14
39	[4OSO3][6OSO3]Galβ1-4GlcNAcβ-Sp0	30	84	729	723
40	[4OSO3]Galβ1-4GlcNAcβ-Sp8	193	116	-28	20
41	6-H_2PO_3Manα–Sp8	215	86	436	240
42	[6OSO3]Galβ1-4Glcβ–Sp0	412	53	38	53
43	[6OSO3]Galβ1-4Glcβ–Sp8	292	60	258	46
44	[6OSO3]Galβ1-4GlcNAcβ–Sp8	321	82	-21	5
45	[6OSO3]Galβ1-4[6OSO3]Glcβ-Sp8	540	181	600	266
46	NeuAcα2-3[6OSO3]Galβ1-4GlcNAcβ–Sp8	239	68	38	41
47	[6OSO3]GlcNAcβ–Sp8	116	54	90	39
48	9NAcNeu5Acα-Sp8	43	18	17882	7296
49	9NAcNeu5Acα2-6Galβ1-4GlcNAcβ-Sp8	199	87	5319	2738
50	Manα1-3(Manα1-6)Manβ1-4GlcNAcβ1-4GlcNAcβ-Sp13	245	102	41	24
51	GlcNAcβ1-2Manα1-3(GlcNAcβ1-2Manα1-6)Manβ1-4GlcNAcβ1-4GlcNAcβ-Sp13	237	144	69	57
52	Galβ1-4GlcNAcβ1-2Manα1-3(Galβ1-4GlcNAcβ1-2Manα1-6)Manβ1-4GlcNAcβ1-4GlcNAcβ-Sp13	0	21	14	9
53	Neu5Acα2-6Galβ1-4GlcNAcβ1-2Manα1-3(Neu5Acα2-6Galβ1-4GlcNAcβ1-2Manα1-6)Manβ1-4GlcNAcβ1-4GlcNAcβ-Sp13	258	59	78	53
54	Neu5Acα2-6Galβ1-4GlcNAcβ1-2Manα1-3(Neu5Acα2-6Galβ1-4GlcNAcβ1-2Manα1-6)Manβ1-4GlcNAcβ1-4GlcNAcβ-Sp8	302	43	496	169
55	Fucα1-2Galβ1-3GalNAcβ1-3Galα-Sp9	123	36	658	430
56	Fucα1-2Galβ1-3GalNAcβ1-3Galα1-4Galβ1-4Glcβ-Sp9	240	77	-11	29
57	Fucα1-2Galβ1-3(Fucα1-4)GlcNAcβ–Sp8	203	36	231	203
58	Fucα1-2Galβ1-3GalNAcα–Sp8	23	5	-16	12
59	Fucα1-2Galβ1-3GalNAcβ1-4(Neu5Acα2-3)Galβ1-4Glcβ-Sp0	133	124	156	110
60	Fucα1-2Galβ1-3GalNAcβ1-4(Neu5Acα2-3)Galβ1-4Glcβ-Sp9	11	18	446	386
61	Fucα1-2Galβ1-3GlcNAcβ1-3Galβ1-4Glcβ–Sp10	81	55	213	124
62	Fucα1-2Galβ1-3GlcNAcβ1-3Galβ1-4Glcβ–Sp8	284	43	193	65
63	Fucα1-2Galβ1-3GlcNAcβ–Sp0	188	55	250	73
64	Fucα1-2Galβ1-3GlcNAcβ–Sp8	76	48	0	13
65	Fucα1-2Galβ1-4(Fucα1-3)GlcNAcβ1-3Galβ1-4(Fucα1-3)GlcNAcβ-Sp0	126	39	42	25
66	Fucα1-2Galβ1-4(Fucα1-3)GlcNAcβ1-3Galβ1-4(Fucα1-3)GlcNAcβ1-3Galβ1-4(Fucα1-3)GlcNAcβ-Sp0	149	57	64	39
67	Fucα1-2Galβ1-4(Fucα1-3)GlcNAcβ–Sp0	111	52	286	159
68	Fucα1-2Galβ1-4(Fucα1-3)GlcNAcβ–Sp8	74	71	37	20
69	Fucα1-2Galβ1-4GlcNAcβ1-3Galβ1-4GlcNAc–Sp0	242	86	1477	692
70	Fucα1-2Galβ1-4GlcNAcβ1-3Galβ1-4GlcNAcβ1-3Galβ1-4GlcNAcβ-Sp0	189	11	-32	9
71	Fucα1-2Galβ1-4GlcNAcβ–Sp0	1011	909	-30	33
72	Fucα1-2Galβ1-4GlcNAcβ–Sp8	24	7	92	76
73	Fucα1-2Galβ1-4Glcβ–Sp0	40	48	82	39
74	Fucα1-2Galβ–Sp8	259	70	-9	18
75	Fucα1-3GlcNAcβ-Sp8	13	20	578	339
76	Fucα1-3GlcNAcβ-Sp8	0	15	30	15
77	Fucα1-4GlcNAcβ–Sp8	49	43	704	414
78	Fucβ1-3GlcNAcβ-Sp8	168	97	33	20

79	GalNAcα1-3(Fucα1-2)Galβ1-3GlcNAcβ-Sp0	44	15	972	693
80	GalNAcα1-3(Fucα1-2)Galβ1-4(Fucα1-3)GlcNAcβ-Sp0	581	279	7	10
81	GalNAcα1-3(Fucα1-2)Galβ1-4GlcNAcβ-Sp0	138	46	-19	11
82	GalNAcα1-3(Fucα1-2)Galβ1-4GlcNAcβ–Sp8	418	322	63	63
83	GalNAcα1-3(Fucα1-2)Galβ1-4Glcβ-Sp0	73	37	66	25
84	GalNAcα1-3(Fucα1-2)Galβ–Sp8	67	46	544	215
85	GalNAcα1-3GalNAcβ–Sp8	48	32	46	29
86	GalNAcα1-3Galβ–Sp8	451	189	490	180
87	GalNAcα1-4(Fucα1-2)Galβ1-4GlcNAcβ-Sp8	137	80	117	29
88	GalNAcβ1-3GalNAcα–Sp8	63	26	734	467
89	GalNAcβ1-3(Fucα1-2)Galβ-Sp8	243	36	31	6
90	GalNAcβ1-3Galα1-4Galβ1-4GlcNAcβ-Sp0	168	50	201	51
91	GalNAcβ1-4(Fucα1-3)GlcNAcβ-Sp0	28	33	28	13
92	GalNAcβ1-4GlcNAcβ–Sp0	127	27	95	43
93	GalNAcβ1-4GlcNAcβ–Sp8	113	53	-30	12
94	Galα1-2Galβ–Sp8	111	17	186	101
95	Galα1-3(Fucα1-2)Galβ1-3GlcNAcβ-Sp0	61	18	101	17
96	Galα1-3(Fucα1-2)Galβ1-4(Fucα1-3)GlcNAcβ-Sp0	91	48	986	985
97	Galα1-3(Fucα1-2)Galβ1-4GlcNAc-Sp0	87	50	138	103
98	Galα1-3(Fucα1-2)Galβ1-4Glcβ-Sp0	-21	9	-25	29
99	Galα1-3(Fucα1-2)Galβ–Sp8	85	7	41	9
100	Galα1-3(Galα1-4)Galβ1-4GlcNAcβ-Sp8	97	24	240	135
101	Galα1-3GalNAcα-Sp8	178	33	23	14
102	Galα1-3GalNAcβ–Sp8	78	38	221	121
103	Galα1-3Galβ1-4(Fucα1-3)GlcNAcβ–Sp8	31	35	50	47
104	Galα1-3Galβ1-3GlcNAcβ-Sp0	62	36	625	182
105	Galα1-3Galβ1-4GlcNAcβ–Sp8	164	48	42	38
106	Galα1-3Galβ1-4Glcβ–Sp0	130	33	1671	1341
107	Galα1-3Galβ–Sp8	222	8	31	47
108	Galα1-4(Fucα1-2)Galβ1-4GlcNAcβ-Sp8	-3	4	3180	1576
109	Galα1-4Galβ1-4GlcNAcβ–Sp0	158	55	-33	10
110	Galα1-4Galβ1-4GlcNAcβ–Sp8	25	12	549	353
111	Galα1-4Galβ1-4Glcβ–Sp0	67	24	56	59
112	Galα1-4GlcNAcβ–Sp8	-14	19	121	59
113	Galα1-6Glcβ-Sp8	147	28	32	23
114	Galβ1-2Galβ–Sp8	300	127	605	321
115	Galβ1-3(Fucα1-4)GlcNAcβ1-3Galβ1-4(Fucα1-3)GlcNAcβ-Sp0	7	16	36	30
116	Galβ1-3(Fucα1-4)GlcNAcβ1-3Galβ1-4GlcNAcβ-Sp0	125	58	612	226
117	Galβ1-3(Fucα1-4)GlcNAc–Sp0	164	84	-23	11
118	Galβ1-3(Fucα1-4)GlcNAc–Sp8	160	107	130	48
119	Galβ1-3(Fucα1-4)GlcNAcβ–Sp8	50	24	25	14
120	Galβ1-3(Galβ1-4GlcNAcβ1-6)GalNAcα-Sp8	9	30	563	310
121	Galβ1-3(GlcNAcβ1-6)GalNAcα-Sp8	27	32	-7	23
122	Galβ1-3(Neu5Acα2-6)GalNAcα-Sp8	-22	18	58	14
123	Galβ1-3(Neu5Acβ2-6)GalNAcα-Sp8	67	42	32	29
124	Galβ1-3(Neu5Acα2-6)GlcNAcβ1-4Galβ1-4Glcβ-Sp10	89	40	494	184
125	Galβ1-3GalNAcα-Sp8	270	72	-20	9
126	Galβ1-3GalNAcβ–Sp8	59	80	503	354
127	Galβ1-3GalNAcβ1-3Galα1-4Galβ1-4Glcβ-Sp0	44	11	14	32
128	Galβ1-3GalNAcβ1-4(Neu5Acα2-3)Galβ1-4Glcβ-Sp0	117	61	594	225
129	Galβ1-3GalNAcβ1-4Galβ1-4Glcβ–Sp8	194	66	12	17
130	Galβ1-3Galβ–Sp8	74	38	517	245
131	Galβ1-3GlcNAcβ1-3Galβ1-4GlcNAcβ-Sp0	149	80	-7	16
132	Galβ1-3GlcNAcβ1-3Galβ1-4Glcβ–Sp10	167	43	172	55
133	Galβ1-3GlcNAcβ–Sp0	120	78	-12	17
134	Galβ1-3GlcNAcβ–Sp8	502	160	875	807
135	Galβ1-4(Fucα1-3)GlcNAcβ–Sp0	36	16	51	42
136	Galβ1-4(Fucα1-3)GlcNAcβ–Sp8	130	126	87	64
137	Galβ1-4(Fucα1-3)GlcNAcβ1-4Galβ1-4(Fucα1-3)GlcNAcβ-Sp0	30	22	52	30
138	Galβ1-4(Fucα1-3)GlcNAcβ1-4Galβ1-4(Fucα1-3)GlcNAcβ1-4Galβ1-4(Fucα1-3)GlcNAcβ–Sp0	191	176	431	237
139	Galβ1-4[6OSO3]Glcβ–Sp0	139	59	1	16
140	Galβ1-4[6OSO3]Glcβ–Sp8	-2	26	537	236
141	Galβ1-4GalNAcα1-3(Fucα1-2)Galβ1-4GlcNAcβ-Sp8	249	81	93	56
142	Galβ1-4GalNAcβ1-3(Fucα1-2)Galβ1-4GlcNAcβ-Sp8	83	9	123	114
143	Neu5Acα2-3Galβ1-4GlcNAcβ1-2Manα1-3(Neu5Acα2-3Galβ1-4GlcNAcβ1-2Manα1-6)Manβ1-4GlcNAcβ1-4GlcNAcβ-Sp12	166	92	30636	5205
144	Galβ1-4GlcNAcβ1-3GalNAcα–Sp8	66	20	89	40
145	Galβ1-4GlcNAcβ1-3Galβ1-4(Fucα1-3)GlcNAcβ1-3Galβ1-4(Fucα1-3)GlcNAcβ-Sp0	251	95	403	180
146	Galβ1-4GlcNAcβ1-3Galβ1-4GlcNAcβ1-3Galβ1-4GlcNAcβ–Sp0	65	35	-4	20
147	Galβ1-4GlcNAcβ1-3Galβ1-4GlcNAcβ–Sp0	728	644	35	15
148	Galβ1-4GlcNAcβ1-3Galβ1-4Glcβ–Sp0	-4	17	54	56
149	Galβ1-4GlcNAcβ1-3Galβ1-4Glcβ–Sp8	204	104	138	18
150	Galβ1-4GlcNAcβ1-6(Galβ1-3)GalNAcα–Sp8	59	22	22	34
151	Galβ1-4GlcNAcβ1-6GalNAcα–Sp8	59	22	63	107
152	Galβ1-4GlcNAcβ–Sp0	58	25	57	15
153	Galβ1-4GlcNAcβ–Sp8	33	24	43	79
154	Galβ1-4Glcβ–Sp0	326	102	28	17
155	Galβ1-4Glcβ–Sp8	270	78	268	89
156	GlcNAcα1-3Galβ1-4GlcNAcβ-Sp8	93	29	7	14
157	GlcNAcα1-6Galβ1-4GlcNAcβ-Sp8	50680	7409	697	285
158	GlcNAcβ1-2Galβ1-3GalNAcα–Sp8	155	68	-10	3
159	GlcNAcβ1-3(GlcNAcβ1-6)GalNAcα–Sp8	131	143	428	45
160	GlcNAcβ1-3(GlcNAcβ1-6)Galβ1-4GlcNAcβ–Sp8	100	46	58	36
161	GlcNAcβ1-3GalNAcα–Sp8	3	7	167	78

162	GlcNAcβ1-3Galβ-Sp8	19	9	1	12
163	GlcNAcβ1-3Galβ1-3GalNAcα-Sp8	40	46	-7	18
164	GlcNAcβ1-3Galβ1-4GlcNAcβ–Sp0	24	11	7	25
165	GlcNAcβ1-3Galβ1-4GlcNAcβ-Sp8	99	12	90	27
166	GlcNAcβ1-3Galβ1-4GlcNAcβ1-3Galβ1-4GlcNAcβ-Sp0	204	74	71	99
167	GlcNAcβ1-3Galβ1-4Glcβ–Sp0	173	39	361	144
168	GlcNAcβ1-4MDPLys	53	31	61	44
169	GlcNAcβ1-4(GlcNAcβ1-6)GalNAcα-Sp8	172	27	95	43
170	GlcNAcβ1-4Galβ1-4GlcNAcβ-Sp8	92	39	-43	10
171	(GlcNAcβ1-4)6β-Sp8	92	62	1948	1928
172	(GlcNAcβ1-4)5β-Sp8	27	17	76	32
173	GlcNAcβ1-4GlcNAcβ1-4GlcNAcβ–Sp8	73	25	16	17
174	GlcNAcβ1-6(Galβ1-3)GalNAcα–Sp8	1	20	1	31
175	GlcNAcβ1-6GalNAcα–Sp8	-15	17	630	663
176	GlcNAcβ1-6Galβ1-4GlcNAcβ-Sp8	84	30	51	37
177	Glcα1-4Glcβ–Sp8	-10	16	602	209
178	Glcα1-4Glca–Sp8	236	24	-1	10
179	Glcα1-6Glcα1-6Glcβ-Sp8	63	22	120	34
180	Glcβ1-4Glcβ-Sp8	14	33	5	11
181	Glcβ1-6Glcβ-Sp8	409	148	29	101
182	G-ol-Sp8	322	55	25	17
183	GlcAa-Sp8	225	103	1099	532
184	GlcAβ-Sp8	182	41	42	22
185	GlcAβ1-3Galβ-Sp8	128	62	1089	585
186	GlcAβ1-6Galβ-Sp8	131	68	33	16
187	KDNα2-3Galβ1-3GlcNAcβ–Sp0	4	24	130	50
188	KDNα2-3Galβ1-4GlcNAcβ–Sp0	57	28	39	14
189	Manα1-2Manα1-2Manα1-3Manα-Sp9	138	149	410	200
190	Manα1-2Manα1-3(Manα1-2Manα1-6)Manα-Sp9	66	22	55	43
191	Manα1-2Manα1-3Manα-Sp9	25	14	801	207
192	Manα1-6(Manα1-2Manα1-3)Manα1-6(Manα2Manα1-3)Manβ1-4GlcNAcβ1-4GlcNAcβ-Sp12	152	55	10	19
193	Manα1-2Manα1-6(Manα1-3)Manα1-6(Manα2Manα2Manα1-3)Manβ1-4GlcNAcβ1-4GlcNAcβ-Sp12	57	25	123	44
194	Manα1-2Manα1-2Manα1-3(Manα1-2Manα1-3(Manα1-2Manα1-6)Manα1-6)Manβ1-4GlcNAcβ1-4GlcNAcβ-Sp12	132	40	22	12
195	Manα1-3(Manα1-6)Manα–Sp9	90	82	340	122
196	Manα1-3(Manα1-2Manα1-2Manα1-6)Manα-Sp9	105	24	-48	8
197	Manα1-6(Manα1-3)Manα1-6(Manα2Manα1-3)Manβ1-4GlcNAcβ1-4GlcNAcβ-Sp12	230	116	239	118
198	Manα1-6(Manα1-3)Manα1-6(Manα1-3)Manβ1-4GlcNAcβ1-4 GlcNAcβ-Sp12	249	205	-36	4
199	Neu5Acα2-6Galβ1-4GlcNAcβ1-2Manα1-3(Neu5Acα2-3Galβ1-4GlcNAcβ1-2Manα1-6)Manβ1-4GlcNAcβ1-4GlcNAcβ-Sp12	585	363	4591	734
200	Manβ1-4GlcNAcβ-Sp0	48	30	647	337
201	Fucα1-3(Galβ1-4)GlcNAcβ1-2Manα1-3(Fucα1-3(Galβ1-4)GlcNAcβ1-2Manα1-6)Manβ1-4GlcNAcβ1-4GlcNAcβ-Sp20	49	27	-18	11
202	Neu5Acα2-3Galβ1-3GalNAcα-Sp8	12	42	38	27
203	NeuAcα2-8NeuAcα2-8NeuAcα2-8NeuAcα2-3(GalNAcβ1-4)Galβ1-4Glcβ-Sp0	149	58	254	94
204	Neu5Acα2-8Neu5Acα2-8Neu5Acα2-3(GalNAcβ1-4)Galβ1-4Glcβ-Sp0	198	17	6	28
205	Neu5Acα2-8Neu5Acα2-8Neu5Acα2-3Galβ1-4Glcβ–Sp0	82	22	459	154
206	Neu5Acα2-8Neu5Acα2-3(GalNAcβ1-4)Galβ1-4Glcβ–Sp0	139	70	-23	9
207	Neu5Acα2-8Neu5Acα2-8Neu5Acα-Sp8	109	45	322	137
208	Neu5Acα2-3(6-O-Su)Galβ1-4(Fucα1-3)GlcNAcβ–Sp8	18	33	42	26
209	Neu5Acα2-3(GalNAcβ1-4)Galβ1-4GlcNAcβ-Sp0	-47	13	760	283
210	Neu5Acα2-3(GalNAcβ1-4)Galβ1-4GlcNAcβ-Sp8	38	30	1701	1587
211	Neu5Acα2-3(GalNAcβ1-4)Galβ1-4Glcβ–Sp0	1108	1114	51	67
212	NeuAcα2-3(NeuAcα2-3Galβ1-3GalNAcβ1-4)Galβ1-4Glcβ-Sp0	1	27	8552	8260
213	Neu5Acα2-3(Neu5Acα2-6)GalNAcα–Sp8	44	24	418	422
214	Neu5Acα2-3GalNAcα–Sp8	84	71	-7	10
215	Neu5Acα2-3GalNAcβ1-4GlcNAcβ-Sp0	-16	18	19	42
216	Neu5Acα2-3Galβ1-3(6OSO3)GlcNAc-Sp8	253	128	12	22
217	Neu5Acα2-3Galβ1-3(Fucα1-4)GlcNAcβ–Sp8	92	6	52	112
218	NeuAcα2-3Galβ1-3(Fucα1-4)GlcNAcβ1-3Galβ1-4(Fucα1-3)GlcNAcβ Sp0	151	25	8	57
219	Neu5Acα2-3Galβ1-3(Neu5Acα2-3Galβ1-4)GlcNAcβ-Sp8	87	38	18985	5184
220	Neu5Acα2-3Galβ1-3[6OSO3]GalNAcα-Sp8	40	18	-7	3
221	Neu5Acα2-3Galβ1-3(Neu5Acα2-6)GalNAcα–Sp8	2507	1622	1528	661
222	Neu5Acα2-3Galβ-Sp8	9	14	68	53
223	NeuAcα2-3Galβ1-3GalNAcβ1-3Galα1-4Galβ1-4Glcβ-Sp0	4	24	-21	34
224	NeuAcα2-3Galβ1-3GlcNAcβ1-3Galβ1-4GlcNAcβ-Sp0	97	36	-5	5
225	Neu5Acα2-3Galβ1-3GlcNAcβ–Sp0	113	48	7	18
226	Neu5Acα2-3Galβ1-3GlcNAcβ–Sp8	497	243	613	484
227	Neu5Acα2-3Galβ1-4[6OSO3]GlcNAcβ-Sp8	522	126	34	24
228	Neu5Acα2-3Galβ1-4(Fucα1-3)(6OSO3)GlcNAcβ–Sp8	68	40	692	252
229	Neu5Acα2-3Galβ1-4(Fucα1-3)GlcNAcβ1-3Galβ1-4(Fucα1-3)GlcNAcβ1-3Galβ1-4(Fucα1-3)GlcNAcβ–Sp0	24	7	-6	32
230	Neu5Acα2-3Galβ1-4(Fucα1-3)GlcNAcβ–Sp0	172	140	138	70
231	Neu5Acα2-3Galβ1-4(Fucα1-3)GlcNAcβ–Sp8	92	35	-4	22
232	Neu5Acα2-3Galβ1-4(Fucα1-3)GlcNAcβ1-3Galβ-Sp8	220	37	1415	422
233	Neu5Acα2-3Galβ1-4(Fucα1-3)GlcNAcβ1-3Galβ1-4GlcNAcβ-Sp8	362	66	94	57
234	Neu5Acα2-3Galβ1-4GlcNAcβ1-3Galβ1-4(Fucα1-3)GlcNAc-Sp0	92	50	632	385
235	Neu5Acα2-3Galβ1-4GlcNAcβ1-3Galβ1-4GlcNAcβ1-3Galβ1-4GlcNAcβ–Sp0	292	68	42	14
236	Neu5Acα2-3Galβ1-4GlcNAcβ–Sp0	29	28	433	159
237	Neu5Acα2-3Galβ1-4GlcNAcβ–Sp8	82	25	811	777
238	Neu5Acα2-3Galβ1-4GlcNAcβ1-3Galβ1-4GlcNAcβ-Sp0	54	21	7170	4094
239	Neu5Acα2-3Galβ1-4Glcβ–Sp0	645	451	274	101
240	Neu5Acα2-3Galβ1-4Glcβ–Sp8	153	101	21	30
241	Galβ1-4GlcNAcβ1-2Manα1-3(Fucα1-3(Galβ1-4)GlcNAcβ1-2Manα1-6)Manβ1-4GlcNAcβ1-4GlcNAcβ-Sp20	72	9	279	205
242	Neu5Acα2-6GalNAcα–Sp8	91	50	32	41
243	Neu5Acα2-6GalNAcβ1-4GlcNAcβ-Sp0	73	20	-11	9
244	Neu5Acα2-6Galβ1-4[6OSO3]GlcNAcβ-Sp8	187	136	531	265

245	Neu5Acα2-6Galβ1-4GlcNAcβ–Sp0	38	27	-10	13
246	Neu5Acα2-6Galβ1-4GlcNAcβ–Sp8	355	202	555	126
247	Neu5Acα2-6Galβ1-4GlcNAcβ1-3Galβ1-4(Fucα1-3)GlcNAcβ1-3Galβ1-4(Fucα1-3)GlcNAcβ-Sp0	108	30	11	25
248	Neu5Acα2-6Galβ1-4GlcNAcβ1-3Galβ1-4GlcNAcβ-Sp0	253	87	337	160
249	Neu5Acα2-6Galβ1-4Glcβ–Sp0	113	29	50	69
250	Neu5Acα2-6Galβ1-4Glcβ–Sp8	10	18	327	150
251	Neu5Acα2-6Galβ–Sp8	214	82	61	86
252	Neu5Acα2-8Neu5Acα-Sp8	-21	8	3731	2206
253	Neu5Acα2-8Neu5Acα2-3Galβ1-4Glcβ–Sp0	36	16	-4	17
254	Neu5Acβ2-6GalNAcα–Sp8	176	69	860	452
255	Neu5Acβ2-6Galβ1-4GlcNAcβ-Sp8	3714	1022	23573	9774
256	Galβ1-4GlcNAcβ1-2Manα1-3(Neu5Acα2-6Galβ1-4GlcNAcβ1-2Manα1-6)Manβ1-4GlcNAcβ1-4GlcNAcβ-Sp21	144	24	56	47
257	Neu5Gca2-3Galβ1-3(Fucα1-4)GlcNAcβ-Sp0	227	65	40	32
258	Neu5Gca2-3Galβ1-3GlcNAcβ-Sp0	254	71	370	188
259	Neu5Gca2-3Galβ1-4(Fucα1-3)GlcNAcβ-Sp0	15	8	-32	20
260	Neu5Gcα2-3Galβ1-4GlcNAcβ–Sp0	83	87	634	355
261	Neu5Gcα2-3Galβ1-4Glcβ–Sp0	47	6	8	16
262	Neu5Gcα2-6GalNAcα–Sp0	235	164	377	222
263	Neu5Gcα2-6Galβ1-4GlcNAcβ–Sp0	245	76	57	46
264	Neu5Gcα–Sp8	129	45	264	91
265	[3OSO3]Galβ1-4(Fucα1-3)(6OSO3)Glc-Sp0	64	36	20	24
266	[3OSO3]Galβ1-4(Fucα1-3)Glc-Sp0	340	33	1357	1253
267	[3OSO3]Galβ1-4[Fucα1-3][6OSO3]GlcNAc-Sp8	25	20	31	13
268	[3OSO3]Galβ1-4[Fucα1-3]GlcNAc-Sp0	312	77	130	143
269	Fucα1-2[6OSO3]Galβ1-4GlcNAc-Sp0	67	49	405	427
270	Fucα1-2Galβ1-4[6OSO3]GlcNAc-Sp8	156	59	-12	13
271	Fucα1-2[6OSO3]Galβ1-4[6OSO3]Glc-Sp0	-62	15	68	41
272	Fucα1-2-(6OSO3)-Galβ1-4Glc-Sp0	3	9	390	318
273	Fucα1-2-Galβ1-4[6OSO3]Glc-Sp0	92	34	60	33
274	Galβ1-3(Fucα1-4)GlcNAcβ1-3Galβ1-3(Fucα1-4)GlcNAcβ-Sp0	87	23	284	87
275	Galβ1-3-(Galβ1-4GlcNacβ1-6)GalNAc-Sp14	44	36	78	63
276	Galβ1-3(GlcNacβ1-6)GalNAc-Sp14	31	28	184	47
277	Galβ1-3-(Neu5Aa2-3Galβ1-4GlcNacβ1-6)GalNAc-Sp14	51	6	2	23
278	Galβ1-3GalNAc-Sp14	97	88	510	266
279	Galβ1-3GlcNAcβ1-3Galβ1-3GlcNAcβ-Sp0	96	47	52	42
280	Galβ1-4[Fucα1-3][6OSO3]GlcNAc-Sp0	210	123	293	131
281	Galβ1-4[Fucα1-3][6OSO3]Glc-Sp0	22	11	22	13
282	Galβ1-4(Fucα1-3)GlcNAcβ1-3Galβ1-3(Fucα1-4)GlcNAcβ-Sp0	69	62	344	159
283	Galβ1-4GlcNAcβ1-3Galβ1-3GlcNAcβ-Sp0	33	29	53	22
284	Neu5Acα2-3Galβ1-3GlcNAcβ1-3Galβ1-3GlcNAcβ-Sp0	60	48	399	345
285	Neu5Acα2-3Galβ1-4GlcNAcβ1-3Galβ1-3GlcNAcβ-Sp0	51	28	14	19
286	[3OSO3]Galβ1-4[6OSO3]GlcNAcβ-Sp0	18	26	162	155
287	[3OSO3][4OSO3]Galβ1-4GlcNacβ-SpSp0	36	23	-37	24
288	[6OSO3]Galβ1-4[6OSO3]GlcNacβ-Sp0	34	25	190	116
289	6-H2PO3Glcβ-Sp10	234	66	-6	15
290	Galα1-3(Fucα1-2)Galβ–Sp18	165	48	1350	720
291	Galα1-3GalNAcα-Sp16	-2	9	34	41
292	Galβ1-3GalNAcα-Sp16	193	86	225	48
293	Galβ1-3(Neu5Acα2-3Galβ1-4(Fucα1-3)GlcNAcβ1-6)GalNAc–Sp14	195	48	170	88
294	Galβ1-3Galβ1-4GlcNAcβ-Sp8	238	154	257	173
295	Galβ1-4GlcNAcβ1-2Manα1-3(Neu5Acα2-6Galβ1-4GlcNAcβ1-2Manα1-6)Manβ1-4GlcNAcβ1-4GlcNAcβ-Sp12	169	54	42	26
296	Galβ1-4GlcNAcβ1-3(Galβ1-4GlcNAcβ1-6)Galβ1-4GlcNAc-Sp0	206	120	594	240
297	Galβ1-4GlcNAcβ1-3(GlcNAcβ1-6)Galβ1-4GlcNAc-Sp0	76	91	39	25
298	Galβ1-4GlcNAcα1-6Galβ1-4GlcNAcβ-Sp0	60	72	90	69
299	Galβ1-4GlcNAcβ1-6Galβ1-4GlcNAcβ-Sp0	29	69	-28	14
300	GalNAcα-Sp15	-23	22	-16	61
301	GalNAcα1-3(Fucα1-2)Galβ–Sp18	398	140	-9	8
302	GalNAcβ1-3Galβ-Sp8	153	83	272	282
303	GlcAβ1-3GlcNAcβ-Sp8	194	67	41	45
304	GlcNAcβ1-2Manα1-3(Neu5Acα2-6Galβ1-4GlcNAcβ1-2Manα1-6)Manβ1-4GlcNAcβ1-4GlcNAcβ-Sp12	39	11	400	137
305	GlcNAcβ1-2Manα1-3(GlcNAcβ1-2Manα1-6)Manβ1-4GlcNAcβ1-4GlcNAcβ-Sp12	153	63	40	21
306	GlcNAcβ1-3Man-Sp10	74	31	81	56
307	GlcNAcβ1-4GlcNAcβ-Sp10	202	60	2	3
308	GlcNAcβ1-4GlcNAcβ-Sp12	12	23	-31	29
309	HOOC(CH3)CH-3-O-GlcNAcβ1-4GlcNAcβ-Sp10	47	26	-25	5
310	Manα1-3(Manα1-6)Manβ1-4GlcNAcβ1-4GlcNAcβ-Sp12	38	65	29	43
311	Manα1-6Manβ-Sp10	21	22	-11	16
312	Manα1-6(Manα1-3)Manα1-6(Manα1-3)Manβ-Sp10	149	85	90	114
313	Manα1-2Manα1-2Manα1-3(Manα1-2Manα1-6(Manα1-3)Manα1-6)Manα-Sp9	373	50	-1	16
314	Manα1-2Manα1-2Manα1-3(Manα1-2Manα1-6(Manα1-2Manα1-3)Manα1-6)Manα-Sp9	67	10	-46	43
315	Neu5Acα2-3Galβ1-3(Neu5Acα2-3Galβ1-4GlcNAcβ1-6)GalNAc–Sp14	34	31	6240	6201
316	Neu5Acα2-3Galβ1-3(Neu5Acα2-6)GalNAc-Sp14	55	33	100	74
317	Neu5Acα2-3Galβ1-3GalNAc–Sp14	46	10	34	11
318	Neu5Acα2-3Galβ1-4GlcNAcβ1-2Manα1-3(Neu5Acα2-6Galβ1-4GlcNAcβ1-2Manα1-6)Manβ1-4GlcNAcβ1-4GlcNAcβ-Sp12	257	59	3263	1911
319	Neu5Acα2-6Galβ1-4GlcNAcβ1-2Manα1-3(Galβ1-4GlcNAcβ1-2Manα1-6)Manβ1-4GlcNAcβ1-4GlcNAcβ-Sp12	357	55	173	32
320	Neu5Acα2-6Galβ1-4GlcNAcβ1-2Manα1-3(GlcNAcβ1-2Manα1-6)Manβ1-4GlcNAcβ1-4GlcNAcβ-Sp12	21	20	108	139
